# Origin of Self-preservation Effect for Hydrate Decomposition: Coupling of Mass and Heat Transfer Resistances

**DOI:** 10.1038/srep14599

**Published:** 2015-10-01

**Authors:** Dongsheng Bai, Diwei Zhang, Xianren Zhang, Guangjin Chen

**Affiliations:** 1 Department of Chemistry, School of Science, Beijing Technology and Business University, Beijing, 100048, P.R. China; 2 State Key Laboratory of Organic-Inorganic Composites, Beijing University of Chemical Technology, Beijing, 100029, P.R. China; 3 State Key Laboratory of Heavy Oil Processing, School of Chemical Engineering, China University of Petroleum, Beijing, 102249, P.R. China

## Abstract

Gas hydrates could show an unexpected high stability at conditions out of thermodynamic equilibrium, which is called the self-preservation effect. The mechanism of the effect for methane hydrates is here investigated via molecular dynamics simulations, in which an *NVT*/*E* method is introduced to represent different levels of heat transfer resistance. Our simulations suggest a coupling between the mass transfer resistance and heat transfer resistance as the driving mechanism for self-preservation effect. We found that the hydrate is initially melted from the interface, and then a solid-like water layer with temperature-dependent structures is formed next to the hydrate interface that exhibits fractal feature, followed by an increase of mass transfer resistance for the diffusion of methane from hydrate region. Furthermore, our results indicate that heat transfer resistance is a more fundamental factor, since it facilitates the formation of the solid-like layer and hence inhibits the further dissociation of the hydrates. The self-preservation effect is found to be enhanced with the increase of pressure and particularly the decrease of temperature. Kinetic equations based on heat balance calculations is also developed to describe the self-preservation effect, which reproduces our simulation results well and provides an association between microscopic and macroscopic properties.

Gas hydrates are a class of inclusion compounds formed by the physically stable interaction between water and guest molecules, in which guest molecules occupy the cages built by hydrogen-bonded water molecules^1^. There are two common types of gas hydrate structures formed in nature: sI with six 5^12^6^2^ cages and two 5^12^ cages, and sII with eight 5^12^6^4^ cages and sixteen 5^12^ cages^2^. Since the high stability below freezing point of water at atmospheric pressure, gas hydrates are expected to be a new medium for energy storage and transport^3^. Comparing with the traditional energy transport methods, utilization of gas hydrate has several advantages: low cost for gas compression or liquefaction, low investment for pipe installation, suitable for high volume transport, and relatively little impact on the environment.

Over the past 2 or 3 decades, many authors^4^,^5^,^6^,^7^,^8^,^9^,^10^,^11^,^12^,^13^,^14^ have studied the melting of gas hydrates at a temperature blow 273 K and an ambient or medium pressure. An anomalous phenomenon was commonly found that some kinds of hydrates can prevent themselves from further decomposing above their melting points but below the melting point for water, and hence show an unexpected stability. This stability under non-equilibrium conditions is thought to be caused by a layer of ice formed when the hydrate melts, and the ice layer coats the hydrate surface to seal it from further dissociation. This effect is called the “self-preservation” of hydrates^6^. Kuhs *et al.*^15^ attributed the self-preservation effect to a diffusion-limited reduction of guest molecules within the surrounding ice layer. Takeya and Ripmeester^16^ suggested that the self-preservation is related to the interaction between guest and water molecules. A two-step dissociation model was first reported by Handa^4^: the destruction of the host lattice followed by the desorption of the guest molecules. Very recently, a consecutive desorption and melting (CDM) model was proposed by Windmeier and Oellrich^17^ in their theoretical study. They suggested that the desorption of guest molecules near the interface is the reason for hydrate decomposition, which leads to the melting of the superheated empty hydrate lattice. Although the above investigations provide important insights into the anomalous preservation effect, the molecular mechanism for this kind of self-preservation effect remains unclear.

Since the temporal and spatial limitations of the monitoring techniques, molecular simulation becomes a powerful method of providing molecular details of hydrate decomposition. By performing molecular dynamics (MD) simulations, Báez *et al.*^18^ and English *et al.*^19^ found that the surface layer of hydrate is composed of partial cages with guest molecules, and the dissociation near the hydrate-fluid interface is roughly in a stepwise manner. Subsequent MD study further indicated the decomposition occur in a heterogeneous layer-by-layer manner^20^. Tse *et al.*^21^ investigated the decomposition process of sI xenon hydrate with MD simulations, and they found the melted water molecules reassemble into solid-like structure near the hydrate surface to block its further decomposition. Considering the heat transfer during the hydrate melted, Baghel *et al.*^22^ performed micro-canonical MD simulations at temperatures higher than 273 K, and fitted their results to the heat balance equations.

In general, the unexpected hydrate stability is presumably ascribed to the formation of the ice layer on hydrate surface that reduces the diffusion of guest molecules^4^,^6^,^15^,^17^, but the origin of the self-preservation effect, i.e. the molecular mechanism that causes the anomalous effect, remains unclear. Molecular simulation studies aforementioned provide the microscopic insights on the decomposition of gas hydrate, however, the self-preservation effect is clearly of distinct origins as it in fact prevents hydrates from decomposing above their melting point. Inspired by molecular simulation studies on hydrate decomposition^18^,^19^,^20^,^21^,^22^, in this work we investigated the mechanism of the self-preservation effect for methane hydrates via molecular dynamics simulations, and the simulation results suggested a coupling between the mass transfer resistance and heat transfer resistance as the driving mechanism for self-preservation effect.

## Results

### The general feature of self-preservation effect

To investigate the molecular origin of the self-preservation effect, in this work a series of *NVT/E* MD simulations at different temperatures and pressures (see Methods section for details) were performed to explore systematically what is responsible for the effect in hydrate melting. Some general characteristics found from our simulations on the hydrate decomposition are discussed below on the aspect of the self-preservation effect.

**Figure 1:**
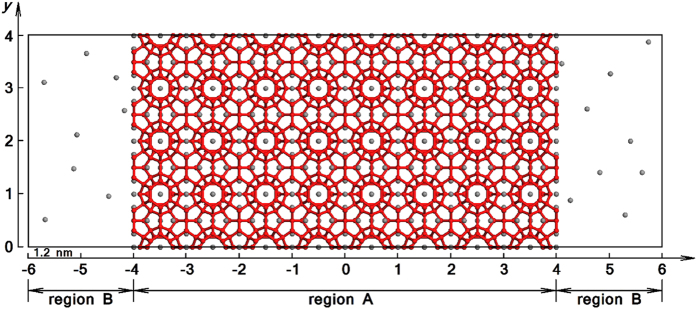
The initial configuration for a typical simulation run. The system was divided into two regions along the *x*-direction: hydrate region (region A) and rest of the system (region B). In the figure, red wire-frame models represent H_2_O molecules and the hydrogen bonds formed between them, while gray spheres represent CH_4_ molecules.

**Figure 2:**
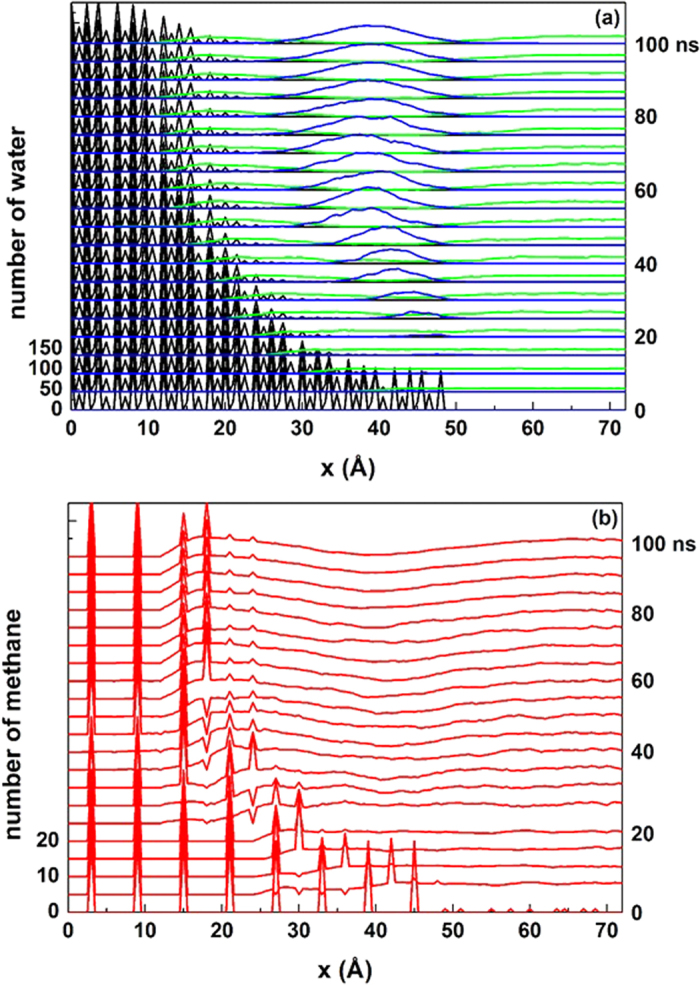
Time evolution of density profile for (**a**) water molecules and (**b**) methane molecules in the system at 265 K and 5 atm. In the figure only the right half part of the system in *x*-direction is shown. In the upper panel, the water molecules belong to hydrate crystal, liquid-like structure and solid-like structure are colored as black, green and blue, respectively.

By taking the *NVT* simulation at 265 K and 5 atm for example (see the initial configuration in Fig. 1), the time evolution of H_2_O and CH_4_ densities during the hydrate decomposition process is given in Fig. 2. It clearly shows that the hydrate was melted to liquid water firstly, and then solid-like water structure was formed and grew continuously on the outer side of the liquid water as the simulation proceeds. No significant resistance for CH_4_ mass transfer is found until a complete solid layer of water is formed, as indicated by the gradual accumulation of CH_4_ between the undecomposed hydrate and the solid-like water layer.

**Figure 3:**
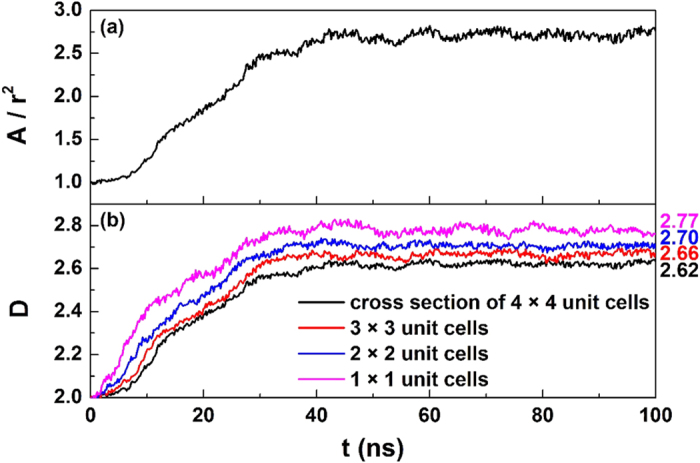
Time evolution of (a) the ratio of surface area *A* to the square of the side length of cross section *r*^2^ and (b) surface fractal dimension for the system at 265 K and 5 atm. In the lower panel, the number on the right side of each curve is the extrapolated value for *t* → ∞.

With the hydrate melted, the surface of hydrate phase is shrinking. Since the hydrate decomposition is not in a layer-by-layer manner, it is difficult to determine the hydrate surface exactly by using the density profile as shown in Fig. 2. Here we used a wave packet method to determine the phase interface: for a water molecule located at 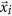, a density profile is given by a wave packet 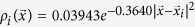 (see Methods section for details). Then, we defined the density profile of the system as the summation of all the wave packets belonging to different water molecules 
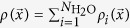
, and the phase interface was determined at 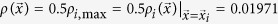. After the determination of the phase interface, surface area can be easily calculated. If the melting surface of hydrate remains flat during the whole process, the surface area is expected to be proportional to the square of the side length. However, Fig. 3a indicates that the surface shows a fractal feature instead. In this work, we assume that the interface area can be written as 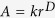, where *k* is a constant, *r* is the side length of cross section and *D* is the surface fractal dimension. The *D* can be determined by fitting above equation to the simulation date, and the results are shown in Fig. 3b. A fractal dimension of *D* ~ 2.62 is obtained for our systems with a cross section of 4 × 4 unit cells. We should note that the size of cross section, however, can affect the fractal feature (Fig. 3b) since fractal structure itself is a nesting structure in different scales^23^.

**Figure 4:**
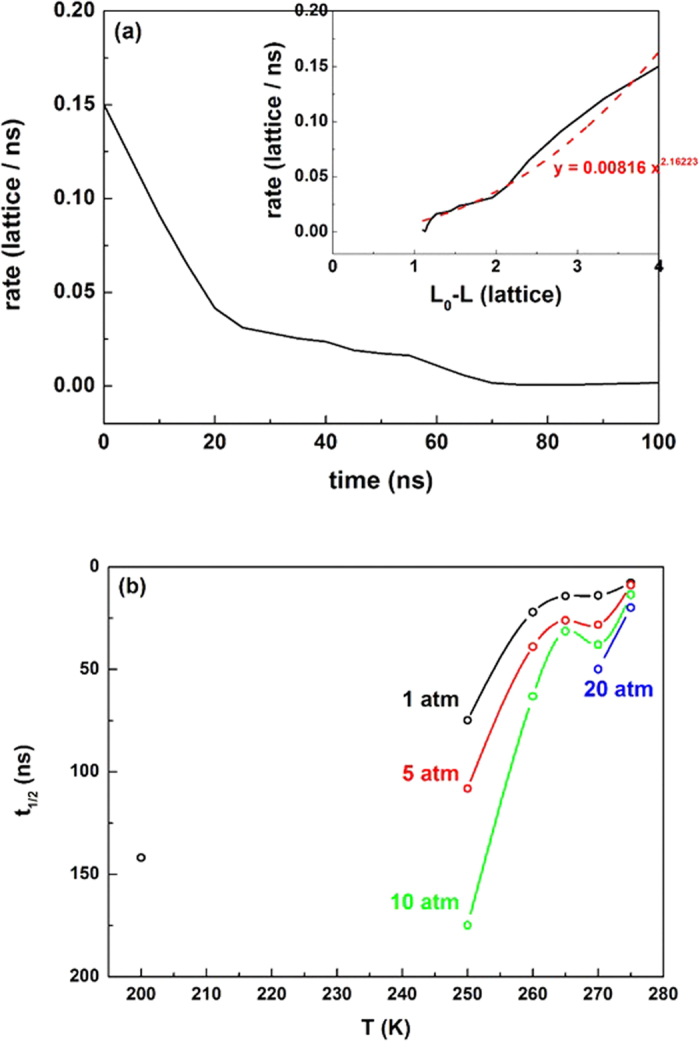
Melting rate and the half-time for hydrate dissociation. (**a**) The time evolution of the melting rate for the system at 265 K and 5 atm. The inset is the fitted rate equation for hydrate dissociation. (**b**) The half-time for hydrate dissociation as function of temperature and pressure.

The occurrence of fractal feature facilitates the decomposition of gas hydrate owing to the obvious increase of surface area. Combined with Figs. 2 and 3, one can find that *D* is greater than 2 although the decomposition slows down and even stops, indicating a high interfacial energy. Therefore, the formation of the solid-like layer results in not only the resistance of mass transfer that increases the chemical potential of methane 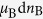 to prevent the hydrate from further dissociation, but also a higher interfacial energy 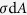 that comes from a fractal surface of melted hydrate. To give the direct evidence of the self-preservation effect, we calculated the time evolution of hydrate melting rate (Fig. 4a). The figure clearly shows that after a short period of rapid melting, the melting slows down gradually and finally stops. The fitted reaction order from the rate equation with respect to hydrate dissociation 
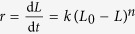
, in which *L* is the actual length and *L*_0_ = 4 is the initial length of hydrate crystal in *x*-direction, shows a value of *n* ~ 2.16 under the thermodynamic conditions (see the inset of Fig. 4a).

### The effect of temperature and pressure

**Figure 5:**
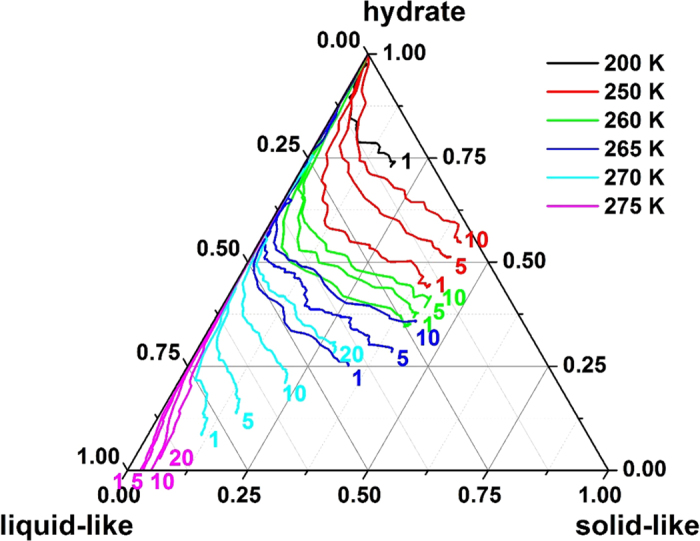
Time evolution trajectories of water components for systems having different temperature and pressure. The number beside each curve is the pressure of the system.

Experimental study shows that natural gas hydrates can be saved more than 1 year at temperature between 255 and 272 K^24^. It is worthy to study how temperature and pressure affect the self-preservation effect on molecular level. During the decomposition process, the component of H_2_O in our simulations (hydrate, liquid-like, and solid-like) was analyzed and the results are shown in a ternary diagram (Fig. 5). The figure clearly indicates that at the end of the totally 100 ns *NVT* simulations, the amount of liquid-like water increases at increasing temperature or at decreasing pressure, indicating a weak self-preservation effect under those conditions. The figure also shows that the solidification of liquid water (i.e., the formation of ice) is more sensitive to temperature than pressure.

**Figure 6:**
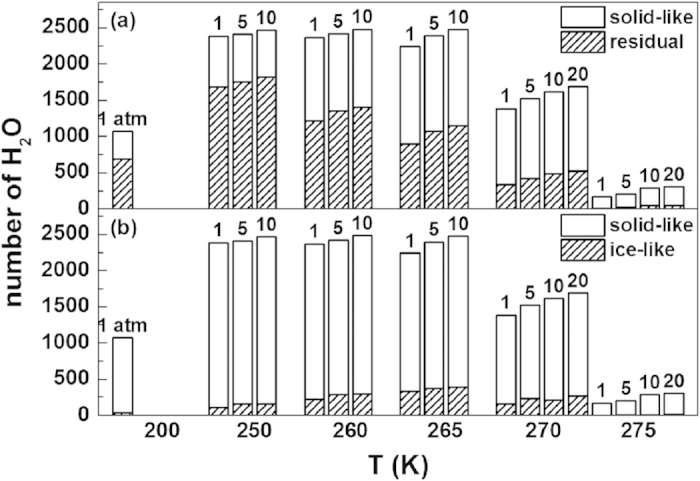
Different origins of the water molecules within the solid-like structures. The total number of solid-like H_2_O molecules, while that of H_2_O molecules in residual rings^25^ (**a**) or in ice-like structure (**b**) within the solid-like structures are both shown. The numbers of each kind of water molecules are counted from the final configuration of all the systems.

The strong self-preservation effect at decreasing temperature or increasing pressure is partly manifested by enhanced memory effect when hydrate melting slows down. By using the topological algorithm developed in our previous study^25^, we calculated the percentage of residual rings in the newly formed solid-like structures, which is shown in Fig. 6a. We note that the residual ring refers to the ring left from the melted hydrate cage, in which the water molecules keep connected each other all the time although the cage is broken. This is the typical feature of memory effect on the microscopic dynamics of hydrate melting. From Fig. 6a, we can see that both the number of solid-like water molecules and the percentage of residual rings within the solid-like layer increase with decreasing temperature, indicating the enhanced memory effect and the enhanced self-preservation effect. Moreover, ice-like water molecules in the solid-like layer were also identified by the algorithm mentioned in Methods section, and the percentage of ice-like water molecules is given in Fig. 6b. Interestingly, the ice motif occurs only at moderate temperature. At high temperature, the melted water molecules are hard to nucleate themselves to form ice, and at low temperature, however, water molecules are also difficult to transform their structure to ice because of their low mobility.

In general, Fig. 6 indicates that at sufficiently high temperature the solid-like structure does not appear, and as the temperature decreases the structure gradually appears near the hydrate surface. Only at a suitable temperature range, in which the memory effect is weakened by the intermediate mobility of water molecules, the solid structure is ice-like. For a deeply cooled system, the structure is amorphous instead. Our simulation results provide a microscopic explanation for the experimental observations^7^,^26^ that the stability of hydrate has a maximum value as temperature changes.

### The effect of heat transfer

**Figure 7:**
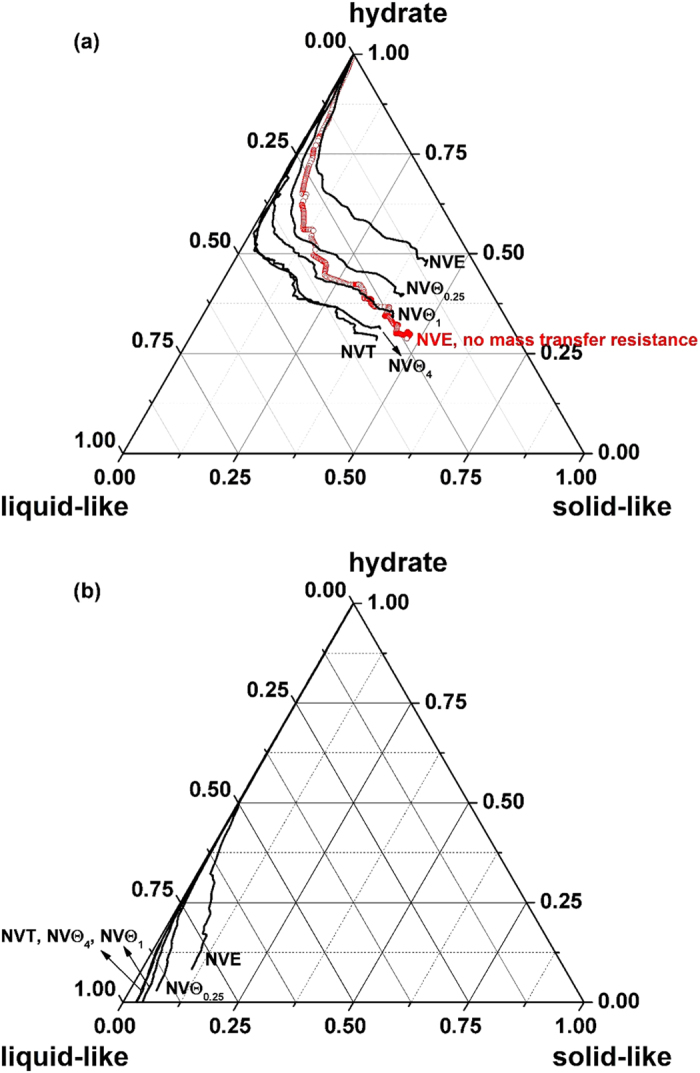
Time evolution trajectories of water components for systems with different heat transfer resistances. (**a**) 265 K and 5 atm and (**b**) 275 K and 1 atm.

Since the latent heat for hydrate decomposition always remove immediately from the thermostat in *NVT* ensemble, it is difficult to discuss the role of heat transfer on the self-preservation effect with *NVT* MD simulations. To illustrate the role the resistance of heat transfer, we performed *NV*Θ simulations instead (see Methods section for details). We still take the system at 265 K and 5 atm as an example, and the time evolution of the component of H_2_O is plotted in Fig. 7a. With decreasing *n* of *NV*Θ_*n*_ simulations, which indicates the enhanced resistance of heat transfer, more melted water molecules show a tendency to form solid-like structure. Thus, the resistance of heat transfer strengthens the self-preservation effect: the resistance of heat transfer facilitates the formation of solid-like layer, which in turn provides the resistance of mass transfer for the diffusion of guest molecules. In contrast, if the resistance of heat transfer was totally remove, as shown in *NVT* simulations at a high temperature of 275 K and 1 atm (Fig. 7b), the hydrate will decompose completely, and only the water molecules in liquid-like structure are left.

To consider the effect of mass transfer, we performed an additional *NVE* simulation at 265 K and 5 atm without the resistance of mass transfer via artificially controlling the released methane: we remove the methane molecules from the system immediately after they are released from the hydrate region during the decomposition process. The initial configuration we used is the same as other simulations, and the simulation time is also 100 ns. To make comparison clearly, the result is also given in Fig. 7a. One can find that without mass transfer resistance, the hydrate melts more thoroughly with less solid-like water molecules in the system. This might be ascribed to the weakened memory effect, since the quickly remove of methane promotes the dissociation of the residual rings. For the same reason, the nucleation and growth of hydrate have been found to begin with adsorption of guest molecules on the faces of pentagonal or hexagonal rings^27^,^28^. Consistently, Jacobson *et al.*^29^ suggested that the adsorbed guest molecules should be considered as a part of the crystal nucleus.

**Figure 8:**
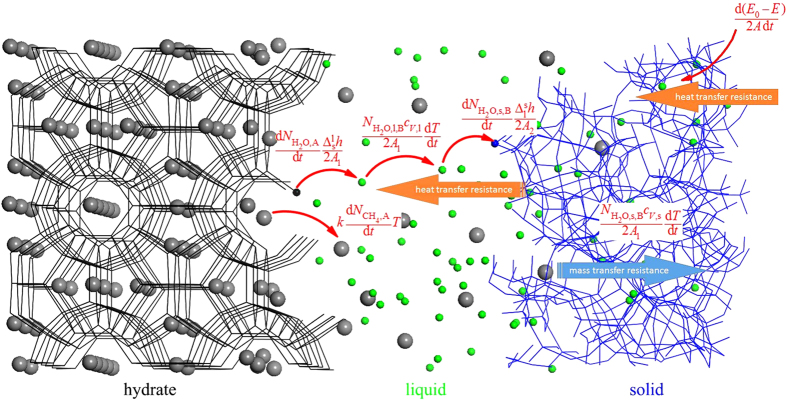
Schematic illustration of the microscopic mechanism for the self-preservation effect. In the figure, water molecules are colored as black, green and blue when they belong to hydrate, liquid and solid, respectively. Methane molecules are modeled as gray spheres.

On the molecular level, the mass transfer resistance comes mainly from the difficulty of methane diffusion. For self-preservation effect, the dynamics is considered as a diffusion-controlled process in which the resistance of mass transfer is necessary. But we emphasize that the fundamental origin for the self-preservation is the resistance of heat transfer, rather than that of mass transfer. The heat transfer resistance induces the formation of solid-like layer, which further enhances the mass transfer resistance. A schematic illustration of the microscopic mechanism is given in Fig. 8.

### Kinetic equations for the self-preservation effect

Based on the heat balance calculations, we developed a kinetic equation to describe the self-preservation effect, which contains contributions from both heat transfer and mass transfer. The information we obtained from the simulations allows us to fit the MD results to the kinetic equations directly. We marked the hydrate region as region A and rest of the system as region B, as is shown in Fig. 1. Note that the range of the two regions varies during a decomposition process.

In an adiabatic *NVE* system, the latent heat of phase change (fusion of hydrate and solidification of melted water) may locally cause the change of system temperature. Hence, a heat balance equation can be written as follows.


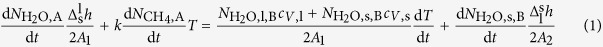


To understand the equation intuitively, a schematic illustration of different terms in the kinetic equation is given in Fig. 8. The two terms on the left side are the latent heat of hydrate melting in region A that includes the hydrate and hydrate-liquid water interface. The first term represents the heat for melting hydrate lattice with *A*_1_ being the fractal surface area for the hydrate-liquid water interface, and the second term is for the heat for desorption of CH_4_ from the phase interface. For the second term, we assumed that the heat needed is a function of the rate of CH_4_ molecules escaped from the hydrate cages 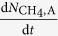
22, i.e., the mass transfer of methane with *k* an arbitrary quantity independent of time. On the right side of the equation, the first term is the exothermic rate in region B far from hydrate and the hydrate-liquid water interface. In region B, we omitted the exothermic rate from methane molecules due to a much smaller number of methane molecules in the region compared to water molecules. Note that the H_2_O-CH_4_ interaction in hydrate-liquid water interface should be included (namely,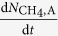 in eq. (1)) due to the enrichment of methane molecules near the interface. The last term of the equation is latent heat of water solidification, with *A*_2_ the fractal area for the interface between the solid-like water and liquid water. Note that the two latent terms in our model are time dependent. Only if the melting rate of hydrate and the freezing rate of liquid water reach zero after initial melting (see Fig. 4a), the two latent heat terms become time independent and correspondingly the equation simplifies to a quasi-stationary equation. In this case, the self-preservation effect dominates the hydrate melting.

We further ignored the difference on structure motif of hydrate crystal and solid-like water, and then we obtain 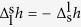. The equation (1) can be rearranged as


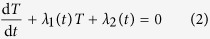


 with 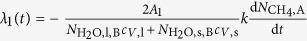, and 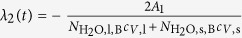

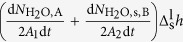
. Combined with the initial condition of 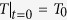, equation (2) can be solved, and the analytical solution is


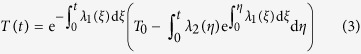


**Figure 9:**
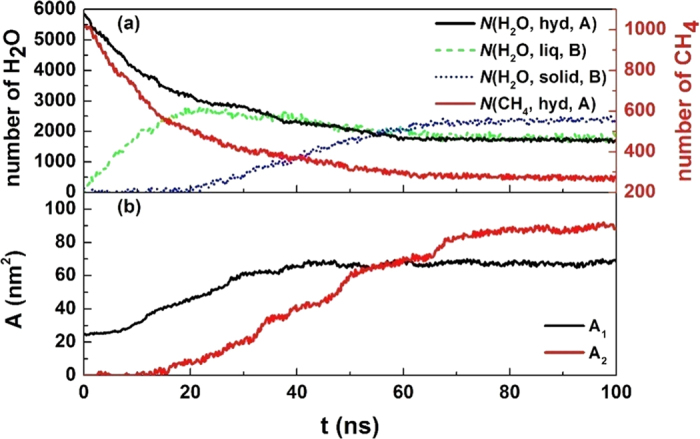
Time evolution of different variables in an *NVE* ensemble simulation at 265 K and 5 atm. (**a**) the number of water and methane molecules belonging to different phases and (**b**) surface fractal dimension.

**Figure 10:**
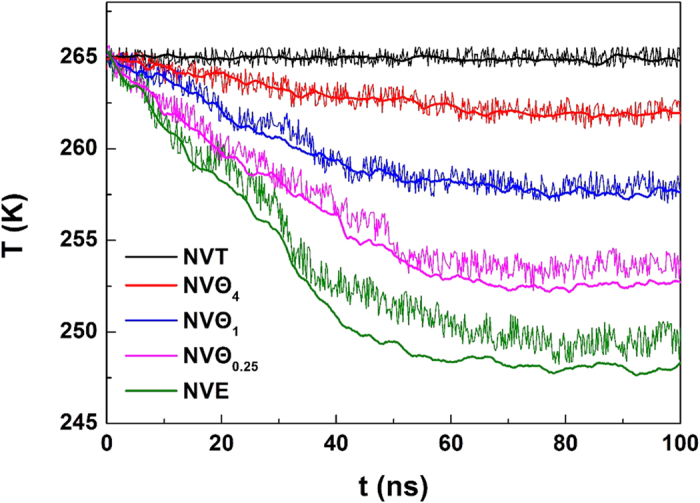
Time evolution of temperature for the system at 265 K and 5 atm. In the figure, the thin lines with strong fluctuation represent the temperature obtained by using the equi-partition theorem, while the thick lines represent that from the equation (3).

In equation (3), we set *c*_*V*,l_ = 75.487 J·mol^−1^·K^−1^, *c*_*V*,s_ = 36.753 J·mol^−1^·K^−1^, and 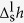 = 6.017 × 10^3^ J·mol^−1^ as in experiment. Time evolution of the number of particles 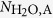, 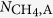,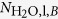 and 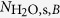, and time evolution of the fractal surface area *A*_1_ (hydrate surface) and *A*_2_ (solid-like water surface) can be obtained from the MD results. As an example, Fig. 9 gives those variables from an *NVE* simulation at 265 K and 5 atm. By integrating the equation (3), we can obtain the temperature of the system versus time (Fig. 10). On the other hand, the temperature can be also calculated from the simulations by using the equi-partition theorem of 
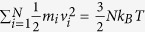
. We can see from Fig. 10 that the temperatures obtained from the two methods are in a good agreement, verifying the correction of the kinetic equation. We note that if we use *r*^2^ instead of the fractal one to represent the interface area, a considerable deviation is observed on the calculated temperature, confirming the fractal feature of the interface.

For *NV*Θ systems, a term of heat flow rate comes from thermostat should be added at the right hand of the heat balance equation (1). Since the energy difference of the system between the initial value *E*_0_ and the current one *E* can be considered as the thermal energy supplied from the environment, the heat flow term can be written as 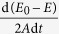, where *A* is heat exchange area and equals to *r*^2^. Hence, the term 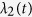 in equation (2) changes to


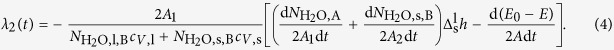


**Figure 11:**
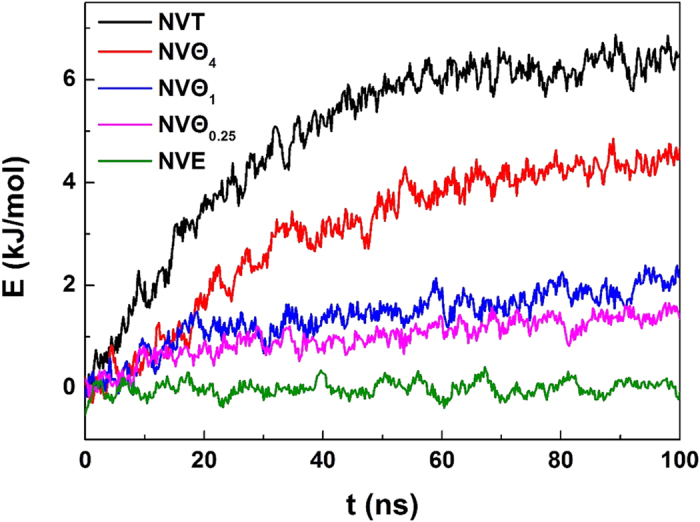
Time evolution of the total energy of the system at 265 K and 5 atm. To facilitate comparison, the energy of the system at t = 0 ns is scaled as 0 kJ/mol.

By providing the time evolution of total energy of the system (Fig. 11), for *NV*Θ systems the temperature can be again obtained from equation (3) (see also Fig. 10). The figure shows that the temperature taken from *NVT* systems is in better agreement than others. A possible interpretation for the deviation might be ascribed to the temperature gradient existed in the *NV*Θ system. Our kinetic equation does not include the spatial distribution of temperature. A more accurate equation should include the contribution from 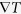.

## Discussion

In this work we investigated the molecular mechanism of the self-preservation effect with a combined *NVT*/*E* MD method, which is in particular designed to include different levels of heat transfer resistance. Our simulations indicate that the methane hydrate was initially melted at the interface, and then the solid-like structure was formed and grew continuously from the melted liquid water, followed by an increase of mass transfer resistance for the diffusion of CH_4_ molecules. This simulation observation is in agreement with the suggestion from experimental studies^30^. More importantly, our simulations indicate a coupling between the mass transfer resistance and heat transfer resistance as the driving mechanism for self-preservation effect.

We found that the phase interface of melting hydrates exhibits fractal characteristics, with a fractal dimension greater than the topological dimension of 2. The fractal characteristics for the phase interface of melting hydrate were also reported experimentally in porous media^23^. Furthermore, our simulations show that the fractal dimension of the interface decreases with increasing cross section (Fig. 3b), indicating that the hydrate crystal in a larger size has a lower interfacial energy and hence shows an enhanced stability. This is in agreement with the stability study for CO_2_ hydrate^13^, which suggested that the hydrate is more stable when it has a larger particle size.

We show that the self-preservation effect is enhanced with decreasing temperature or increasing pressure, and more sensitive to temperature. To compare directly with experimental results, we calculated the half-time of the hydrate dissociation, 
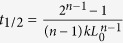
, by using the fitted time evolution of the melting rate (e.g., see the inset of Fig. 4a), and the results are given in Fig. 4b. The variation trend of *t*_1/2_ versus *T* and *p* is in a good agreement with experimental studies^31^, in which the authors similarly showed the anomalous preservation effect at 242–271 K. Note that the order of magnitude of *t*_1/2_ in our work is obviously less than that for the experiments since we only considered the melting of hydrates in a thickness of two lattices. According to our simulations the preservation effect is caused by a combined effects of heat transfer and mass transfer.

Moreover, our simulations indicate that the ice-like motif was observed only at moderate temperature range (Fig. 6), as demonstrated by experimental studies^7^,^26^,^32^. Note that at the temperature below 200 K, the Ic ice structure has not been observed here as in experimental measurement^14^,^33^,^34^, and an amorphous structure for the newly formed solid layer is formed instead. This may be partly due to the much short time scale covered by our simulations.

Our simulations also suggested a coupling between the mass transfer resistance and heat transfer resistance that induces the self-preservation effect. Furthermore, the heat transfer resistance is a more fundamental factor to control the self-preservation effect, in comparison with the mass transfer resistance. This is partly because the solidification of the melted water, which is the first step for the occurrence of self-preservation effect, is caused mainly by the release of latent heat rather than the mass transfer. The heat transfer resistance promotes the formation of solid-like layer, which in turn enhances the resistance of mass transfer. We note that the solid-like layer is formed from the melted liquid water rather than from the hydrate directly because of the considerably higher energy barrier for the direct hydrate-to-ice transition (Figs. 2 and 5). This observation is in a good agreement with the suggestion from the experimental study^35^, in which the authors found that the hydrate always melts to liquid water before solidification, and never undergoes a solid-solid phase transformation.

Finally, based on the heat balance calculations we developed here a kinetic equation, which contains both contributions from heat transfer and mass transfer, to describe the self-preservation effect. The equation provides a method to make association between the molecular-level knowledge and the macroscopic information.

## Methods

### Model and simulation procedure

The MD simulations implemented in LAMMPS^36^ were performed in this work. To obtain the initial configuration, we firstly prepared a perfect sI CH_4_ hydrate and placed it into a simulation box with a size of 4 × 4 × 4 unit cells (1 unit cell equals to 1.245 nm). For the structure of the hydrate, the positions of the oxygen atom in H_2_O molecules and carbon in CH_4_ were obtained from the data of the space group of the crystal structure^37^. This hydrate crystal was relaxed for 2 ns through an *NpT* process at 260 K and 5 atm. Then, we replicated the crystal along the *x*-direction to get an 8 × 4 × 4 repetition (totally 5888 H_2_O molecules and 1024 CH_4_ molecules), and a vacuum layer of 2 × 4 × 4 unit cells was introduced along the *x*-direction to each of the two hydrate interfaces, as is shown in Fig. 1. To investigate the effect of pressure on hydrate decomposition, we added a certain amount of CH_4_ molecules into the vacuum layer to reach the specified value of the pressure of CH_4_ gas (e.g. 5 atm). The amount of CH_4_ added is summarized in Table 1, which is determined by the equation of state developed by Vennix and Kobayashi^38^.

In our simulations, the TIP4P water model^39^ was used and the rigidity was implemented with SHAKE algorithm^40^, while a single point model^41^ was used to model CH_4_ molecules. The unlike parameters of Lennard-Jones potentials were obtained by the Lorentz-Berthelot mixing rule. A cutoff radius of 12 Å was utilized for the short-ranged (Lennard-Jones) interactions, while the long-ranged (Coulomb) interactions were evaluated by using the pppm algorithm^42^. Periodic boundary conditions were imposed in all the three Cartesian directions.

Since the hydrate decomposition involves dissociation and rearrangement of the hydrogen bonds at the microscopic scale, it is usually not considered as an isothermal process. The hydrate decomposition is endothermic^43^, which will cause a drop in temperature^20^ during the melting process. If the heat is supplied from the outside when hydrate melted, however, the temperature drop will be weakened. To investigate the effect of the heat transfer on the self-preservation effect, we performed both *NVT* simulations (closed system), *NVE* simulations (isolated system), and several mixed *NVT*/*E* simulations at different temperature and pressure conditions (see Table 1). The *NVT* simulations fail to consider heat transfer resistance, as the heat needed by the hydrate decomposition can be supplied immediately. In other words, the thermal effect of phase transition is not considered in *NVT* ensemble. To the contrary, *NVE* simulations represent a maximal resistance for heat transfer.

In this work, *NVT* simulations were performed by using the Nosé-Hoover algorithm^44^ and with the relaxation parameter of 0.1 ps; while *NVE* simulations were performed directly. For the simulations of mixed *NVT*/*E*, a series of *NVT* and *NVE* simulations were conducted alternatively. By adjusting the ratio of the steps of the two alternative runs, we can obtain different resistance for heat transfer. The number of steps we used for different mixed *NVT*/*E* runs were listed in Table 2. To simplify the description, we introduce a uniform symbol of *NV*Θ_*n*_ to describe the different ensemble, in which the subscript *n* indicates the step number ratio of *NVT* and *NVE* within a cycle. Therefore, an *NVT* simulation can be written as *NV*Θ_∞_; through a series of mixed *NVT*/*E* marked as *NV*Θ_4_, *NV*Θ_1_ and *NV*Θ_0.25_ with increasing resistance for heat transfer, we finally get *NV*Θ_0_ for *NVE*. With the increase of *n*, the resistance of heat transfer is decreased. A timestep of 1 fs was applied in all our simulations, and all of the *NV*Θ simulations were performed on the timescale of 100 ns.

### Identification of the ice

Because the structure of the solid-like layer formed at the hydrate surface is an important issue, a meticulous analysis scheme should be developed. Although the widely used *F*_4_ and *Q*_6_ order parameters^45^,^46^ can identify ice clusters from its environments, it is difficult to show the topological information about the ice structure.

**Figure 12:**
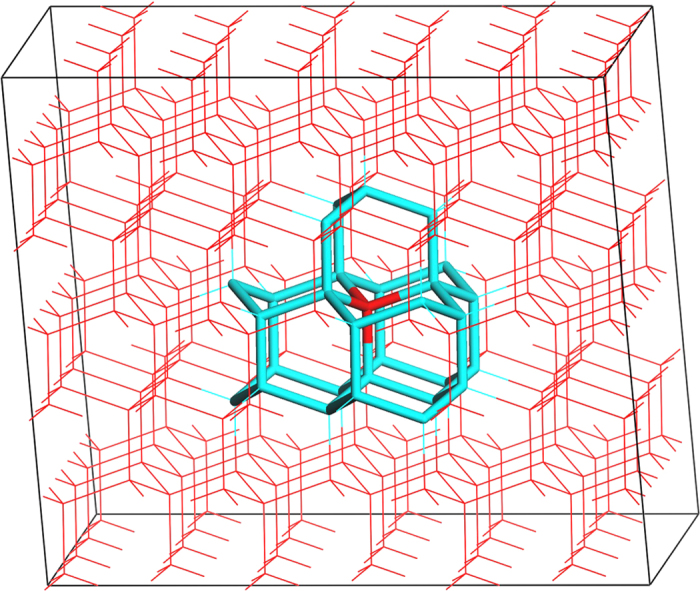
The vertices in the ice Ih crystals. The center vertex of water molecule is shown in red, and the cages associated with it are colored as cyan.

Here we introduced a vertex perception algorithm to obtain ice domains. In ice crystals, each water molecule is tetra-coordinated to four other water molecules by hydrogen bonds, which means a water vertex shared by four polyhedral cages. For both Ih and Ic structure, one water molecule may be involved in (i) one 6^3^ cage (all the three hexagons are chair conformations) and three 6^5^ cages (two are chair and three are boat conformations); or equivalently involved in (ii) six groups of totally twelve six-membered rings (there are three boat-boat groups and three boat-chair groups), as is shown in Fig. 12. In the present work, the melted water molecules are firstly classified into liquid-like or solid-like by their mean square displacement (MSD). Then, we use the ring perception algorithm^47^ and the cage identification algorithm^48^ to search the hexagonal structures and the cage structures within the solid-like water molecules. Different from what we did in hydrate phase, the cages in ice are concave polyhedrons. If a water molecule satisfies any of the above conditions (i) and (ii), it will be considered as an ice-like water molecule.

### A wave packet method to determine the phase interface

Since the hydrate melting does not take place in a layer-by-layer manner, we developed a wave packet method to determine the phase interface. The advantage of this method is that compared with the *x*-density profile method, it can give more details about the shape of the interface.

Firstly, we used a wave packet 
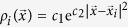
 to expand the density profile of a water molecule located at 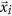 from a scattered density profile 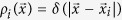, which is not convenient for mathematical analysis. The parameters of *c*_1_ and *c*_2_ were determined with following assumptions: (i) For a water molecule located at 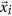, we assume that the density profile at 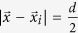 decreases to *λ* (*λ* < 1) times of its maximum density, namely,


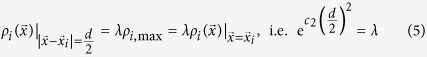


(ii) The integration of density profile 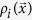 over the whole space should be equal to 1,


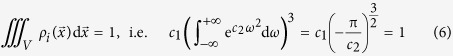


Combining equations (5) and (6), we got


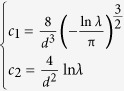
 and 
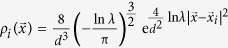
. For the hydrate system, we chose *d* = 2.76 Å (the average distance between neighboring water molecules) and *λ* = 0.5, and thus obtained 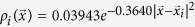.

## Additional Information

**How to cite this article**: Bai, D. *et al.* Origin of Self-preservation Effect for Hydrate Decomposition: Coupling of Mass and Heat Transfer Resistances. *Sci. Rep.* **5**, 14599; doi: 10.1038/srep14599 (2015).

## Figures and Tables

**Table 1:**
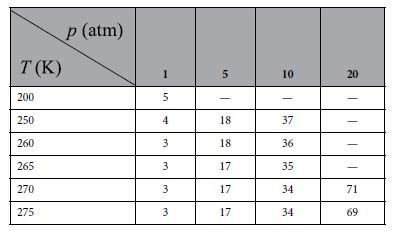
The amount of CH_4_ molecules added in the vacuum layer of the initial configurations.

Note: the symbol ‘—’ in the table represented the conditions we did not simulate because those correspond to thermodynamically stable conditions for CH_4_ hydrates^1^.

**Table 2:** The number of the *NVT* and *NVE* steps within one cycle of the *NV*Θ simulation.

	number of *NVT*steps	number of *NVE*steps
*NV*Θ_∞_(=*NVT*)	1000	0
*NV*Θ_4_	800	200
*NV*Θ_1_	500	500
*NV*Θ_0.25_	200	800
*NV*Θ_0_(=*NVE*)	0	1000
